# One-Pot Synthesis of Organic-Sulfur-Zinc Hybrid Materials via Polycondensation of a Zinc Salt and Thiols Generated *in Situ* from Cyclic Dithiocarbonates

**DOI:** 10.3390/molecules200815049

**Published:** 2015-08-17

**Authors:** Bungo Ochiai, Hirohisa Konta

**Affiliations:** Department of Chemistry and Chemical Engineering, Faculty of Engineering, Yamagata University, 4-3-16 Jonan, Yonezawa, Yamagata 992-8510, Japan; E-Mail: hiro_knt_4290@yahoo.co.jp

**Keywords:** organic-inorganic hybrid, nanoparticle, refractive index, sulfur, zinc, thiol, polycondensation, ring-opening

## Abstract

Soluble organic-sulfur-zinc hybrid polymers were prepared via a one-pot reaction consisting of ring-opening addition and subsequent polycondensation. The first reaction is the nucleophilic ring-opening addition of 2-ethylhexylamine to multifunctional cyclic dithiocarbonates giving multiple thiols *in situ*. The sequential polycondensation of the *in situ* generated thiols with Zn(OAc)_2_ gave the target hybrid polymers. This one-pot method enabled the use of a shorter amine than the previous polycondensation of Zn(OAc)_2_ and purified thiols, which required octadecylamine to obtain a soluble product. The obtained hybrid polymers may be cast as composite films with polystyrene and poly(methyl methacrylate). Owing to the shorter alkyl chain, the calculated *n*_D_ values of the products (1.60 or 1.61) are higher than that of the previous product bearing octadecyl chains (1.53).

## 1. Introduction

High refractive materials are important components for displays, lens, solar cells, and luminescent devices [1,2,3,4,5,6,7,8,9,10,11]. Organic materials are advantageous due to their easy processing and flexibility, but their refractive and mechanical properties are typically inferior to those of inorganic materials. From this point of view, organic-inorganic hybrids have also been explored to fabricate materials taking advantages of the properties of both components [5,6,7,8,9,10,11]. Typical methods are mixing of inorganic ingredients in organic polymers and sol-gel methods. Although both methods yield various refractive materials, the poor affinity between organic and inorganic structures limits the molecular design options. Hybrid materials based on covalent linkages between organic and inorganic structures were especially limited to silane-, germane-, and tin-based hybrids, despite the advantage of acquiring homogeneous materials [10].

As a new design for organic-inorganic hybrid materials from other metals with low affinity with organic structures, we have focused on sulfur as a bridge between organic and inorganic structures. A refractive polymer was obtained via polycondensation of Zn(OAc)_2_ and a thiol prepared by the reaction of octadecylamine and a trifunctional five-membered cyclic dithiocarbonate (1,3,5-tris(2-thioxo-1,3-oxathiolan-5-yl)methyl)-1,3,5-triazinane-2,4,6-trione (TDT)) [11]. The zinc center has two covalent Zn–S linkages, and is ligated by the C=S group. This material may be dispersed in poly(methyl methacrylate) (PMMA), and increased the refractive indices of the composite films. However, the *n*_D_ value calculated for this material is not so high (*n*_D_ calculated from the dependence of the *n*_D_ of the films on the weight ratio was 1.58), probably due to the long octadecyl chains having poor refractive properties. Although we tried to shorten the alkyl chains using benzylamine, *n*-hexylamine, isoamylamine, and 2-ethylhexylamine (EHA), all the products were insoluble in common organic solvents. Plausible reasons are insufficient flexibility to maintain the solubility with these shorter chains and the poor stability of the thiols cross-linked via the oxidative coupling of the thiol moieties giving disulfide linkages. This undesirable oxidative coupling obviously took place with the thiol obtained from EHA that easily turned into solid from viscous oil.

As a method to avoid the oxidative coupling of thiols, we reported one-pot reactions of *in situ* generated multiple thiols from dithiocarbonates and amines for fabrication of well-defined polymers [12,13]. This reaction giving thiols is a *click* reaction [12,13,14,15,16,17,18,19] selective enough to provide a polymer-bearing thiol moieties in the repeating units serving as a polymeric chain transfer agent [12] and dithiols for three-component polyaddition with quantitative atom-economy [13]. We applied this concept to this synthesis of organic-sulfur-zinc hybrid materials. In this paper, we demonstrate that the one-pot procedure may provide soluble hybrid materials with higher refractive indices than the previous material and the resulting materials serve as refractive ingredients for common optical polymers.

## 2. Results and Discussion

### 2.1. Polycondensation of Zn(OAc)_2_ with in Situ Generated Trithiol

**Scheme 1:**
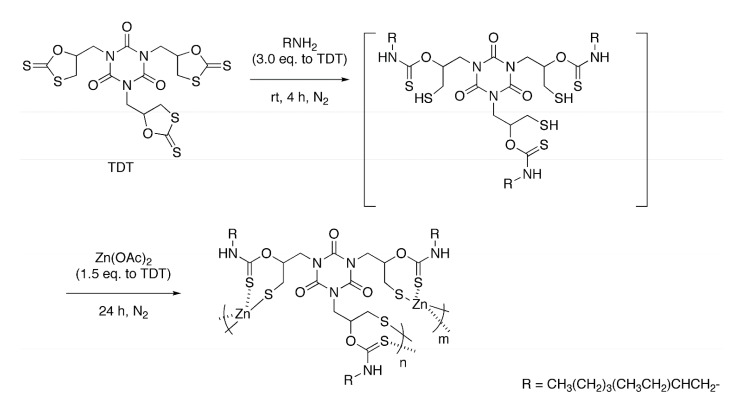
One-pot synthesis of organic-sulfur-zinc hybrid via sequential ring-opening addition of EHA and TDT and polycondensation of the *in situ* generated thiol with Zn(OAc)_2_.

**Figure 1:**
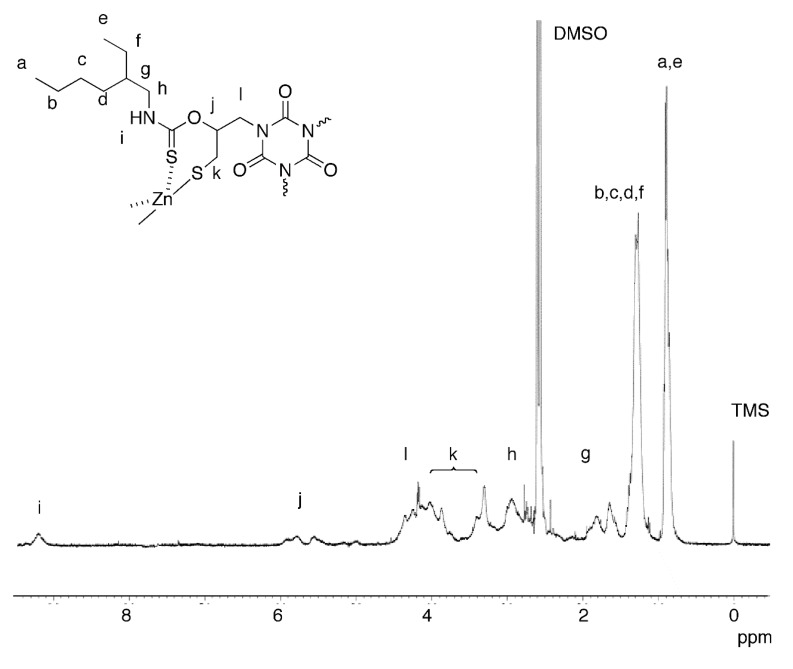
^1^H-NMR spectrum (400 MHz, 5:1 DMSO-*d*_6_/CF_3_COOH) of the polymer obtained in run 1 in Table 1.

We selected EHA as the amine for this reaction, because of the shorter chain and the flexibility originating from the branched structure. TDT and EHA were reacted for 4 h at room temperature under a nitrogen atmosphere, and the corresponding thiol was quantitatively produced *in situ* via a nucleophilic ring-opening of the cyclic dithiocarbonate structure as reported. Then, Zn(OAc)_2_ (1.5 equivalents with respect to SH) in methanol was added, and reacted with the thiol generated *in situ* for 24 h under a nitrogen atmosphere (Scheme 1, Table 1). For example, the reaction in DMSO with the polycondensation at 60 °C gave a corresponding polymer in a 45% yield (run 1). Whereas the product produced via the previous polycondensation using the purified thiol was insoluble in any common solvents [11], the products in this one-pot method were soluble in THF, DMSO, and DMF. This improved solubility may be ascribed to the suppressed cross-linking with oxidative coupling of the thiol moieties. The ^1^H-NMR and IR spectra were analogous to those of the soluble polymer obtained by the previous method. For example, the IR absorptions of the C=S bonds were observed at 1172 and 1168 cm^−1^ for the isolated thiol monomer and the polymer obtained by this one-pot method, respectively, indicating the ligation of the C=S moieties onto the zinc atom [11]. The ^1^H-NMR spectrum of the polymer obtained in run 1 is shown in Figure 1 with the peak assignments. Signals assignable to the 2-ethylhexyl groups, the ring-opened structure originated from TDT, and the NH moieties were observed. The integral ratio of the signals assigned to the two methyl protons in the 2-ethylhexyl groups to that assigned to the methyne proton in the ring-opened unit is 6.0/0.9, which almost agree with the theoretical value (6/1). Other signals were overlapped, and detailed calculation was difficult.

Various conditions were investigated for optimization. First, the effect of solvents was examined using DMSO, THF, and DMF (runs 1–3). The yields were higher with polar DMSO and DMF, and the molecular weight became highest with DMSO. The atom ratios of Zn/S were estimated by energy dispersive X-ray (EDX) analysis, and those of the products obtained in DMSO and THF almost agreed with the theoretical value (0.25). The Zn/S may contain 20% to 30% of errors in a similar manner with the previous products. This result indicates that DMSO is the best solvent among the examined solvents. Then, the temperature for the polycondensation process was varied. As the temperature increases, the polydispersity indices (*M*_w_/*M*_n_) became broadened, probably due to the cross-linking reaction, although the yields and the Zn/S ratios were identical (runs 4 and 5). We also examined the reaction using an excess amount of EHA for neutralization of the eliminated acetic acid to tilt the equilibrium to the product (run 6). As a result, the yield was improved, but the product became partially insoluble in THF. We presumed that the lower solubility originated from the accelerated oxidative coupling of the thiol moieties under basic conditions. These experiments show the appropriate conditions using DMSO as the solvent and the equivalent amount of EHA to the cyclic dithiocarbonate moieties and polycondensation at moderate 30 °C.

**Table 1:** One-pot synthesis of organic-sulfur-zinc hybrid via sequential ring-opening addition of EHA and TDT and polycondensation of the *in situ* generated thiol with Zn(OAc)_2_.

Run	Solvent	Temperature (°C)	R-NH_2_ (Equivalent to TDT)	Yield (%) ^a^	*M*_n_ (*M*_w_/*M*_n_) ^b^	Zn/S ^c^
1	DMSO	30	3.0	45	5800 (1.4)	0.29
2	THF	30	3.0	31	2300 (1.4)	0.31
3	DMF	30	3.0	42	2000 (1.4)	0.15
4	DMSO	40	3.0	45	2300 (5.7)	0.32
5	DMSO	60	3.0	47	4000 (39)	0.35
6	DMSO	30	6.0	56	8400 (1.6) ^d^	0.34

Conditions: TDT = 0.2 mmol; Zn(OAc)_2_ = 0.3 mmol; solvent = 5 mL; N_2_; 1st step, rt, 4 h; 2nd step, 24 h. ^a^ Isolated yield after precipitation to methanol; ^b^ Estimated by SEC (THF, polystylene standard); ^c^ Estimated by EDX (Ratios calculated as averages of 10 spots); ^d^ THF soluble part.

### 2.2. Polycondensation of Zn(OAc)_2_ with in Situ Generated Dithiol

**Scheme 2:**
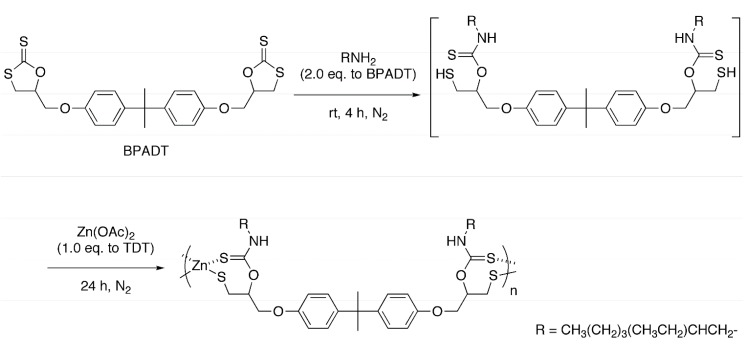
One-pot synthesis of organic-sulfur-zinc hybrid via sequential ring-opening addition of EHA and BPADT and polycondensation of the *in situ* generated thiol with Zn(OAc)_2_.

**Figure 2:**
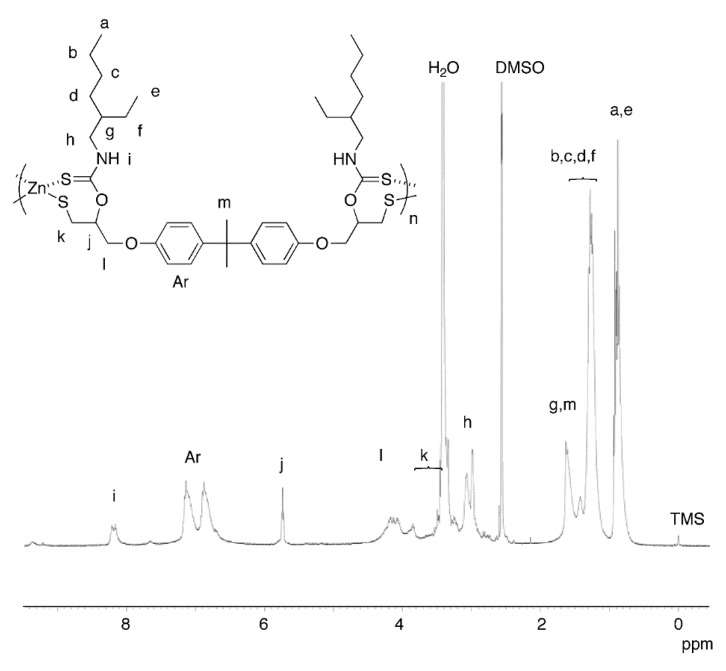
^1^H-NMR spectrum (400 MHz, DMSO*-d*_6_) of the polymer obtained in run 1 in Table 2.

The reaction employing a bifunctional cyclic dithiocarbonate obtained from bisphenol-A diglycidyl ether (BPADT) was also examined, because the aforementioned trifunctional monomer may lead to networked structures decreasing the solubility. The reaction was conducted under the identical conditions with run 1 in Table 1 (Scheme 2, Table 2). As a result, a soluble polymer (*M*_n_ = 6600, *M*_w_/*M*_n_ = 1.3) was obtained in a 59% yield (run 1). The ^1^H-NMR spectrum is in agreement with the plausible structure as indicated in Figure 2, in a similar manner to the polymer obtained from TDT. The conditions for this polycondensation were also optimized in a similar manner. The products obtained in DMSO and DMF (run 3) were soluble in THF and DMF, but the product obtained in THF was insoluble in any common organic solvents (run 2). The best solvent was DMSO because of the highest yield and the molecular weight. The reactions at a higher temperature (run 5) and with an excess amount of EHA (run 6) were unsuccessful as well. Judging from the insoluble nature of the product obtained in THF (run 2), the solubility of the products from BPADT was lower than that from TDT despite the initial presumption that the solubility would be improved with replacing the trifunctional monomer with the bifunctional monomer. Although the reason is unclear, we reached to a presumption that zinc oxide structure might also be produced by the hydrolysis of the terminal Zn–OAc structure, from the high Zn/S values. However, the obtained products are amorphous, and we could not find proofs for zinc oxide structures.

**Table 2:** One-pot synthesis of organic-sulfur-zinc hybrid via sequential ring-opening addition of EHA and BPADT and polycondensation of the *in situ* generated thiol with Zn(OAc)_2_.

Run	Solvent	Temperature (°C)	R-NH_2_ (Equivalent to C=S)	Yield (%) ^a^	*M*_n_ (*M*_w_/*M*_n_) ^b^	Zn/S ^c^
1	DMSO	30	3.0	59	6600 (1.3)	0.53
2	THF	30	3.0	39	Insoluble	0.48
3	DMF	30	3.0	58	1300 (1.4)	0.40
4	DMSO	60	3.0	28	3200 (1.7)	0.44
5	DMSO	30	6.0	63	Insoluble	0.39

Conditions: BPA5DT = 0.2 mmol, Zn(OAc)_2_ = 0.2 mmol, Solvent = 5 mL, N_2_; 1st step, rt, 4 h; 2nd step, 24 h. ^a^ Isolated yield after precipitation to methanol; ^b^ Estimated by SEC (THF, polystylene standard); ^c^ Estimated by EDX (Ratios calculated as averages of 10 spots).

### 2.3. Properties of Organic-Sulfur-Zinc Hybrid Materials Obtained by the One-Pot Method

#### 2.3.1. Films with PMMA and PS

**Figure 3:**
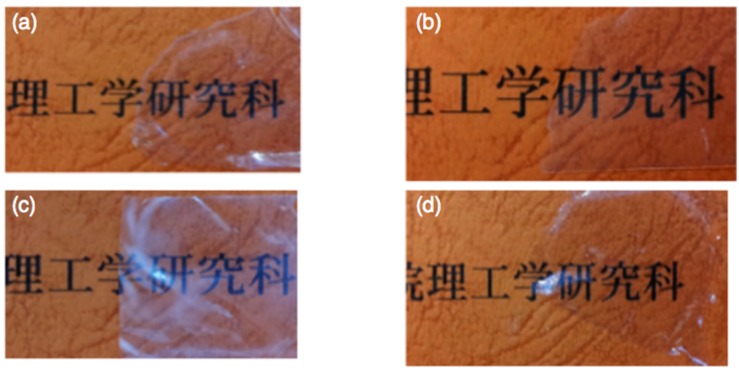
Photo images of composite films of the zinc hybrid polymers with common polymers, (**a**) TZn/PMMA; (**b**) TZn/PS; (**c**) BZn/PMMA; and (**d**) BZn/PS.

For the application as refractive ingredients, we prepared composite films of the obtained hybrid polymers with PMMA and polystyrene (PS). We employed the hybrid polymers obtained in DMSO at 30 °C (abbreviated as TZn and BZn). The hybrid polymers were mixed with the polymers at a weight ratio of 10:90, and then dissolved in THF. The solutions were cast on a Teflon mold, and THF was evaporated off at room temperature. Composite films were obtained after drying under reduced pressure. The composite films of TZn/PS, BZn/PS, and TZn/PMMA were transparent, but the film of BZn/PMMA was opaque (Figure 3). The films from PMMA were flexible, but those from PS were brittle and the films were easily broken.

The thermal properties of the composite films were evaluated with thermogravimetry analysis (TGA) and differential scanning calorimetry (DSC) (Table 3). The low thermal stability of the zinc hybrid polymers, due to the thermally unstable thiourethane moieties with the N-H structure [20], unfortunately decreased the thermal stability. However, the 5-wt % weight loss temperature (*T*_d5_) of BZn/PS was higher than those of other composites.

**Table 3:** Thermal behaviors of the zinc hybrid polymers and their composite films with common polymers.

	BZn	BZn_10_/PS_90_	BZn_10_/PMMA_90_	TZn	TZn_10_/PS_90_	TZn_10_/PMMA_90_	PS	PMMA
*T*_d5_ (°C) ^a^	192	272	161	170	113	139	380	281
*T*_g_ (°C) ^b^	40	86	– ^c^	– ^c^	– ^c^	– ^c^	100	105

^a^ 5-wt % weight loss temperatures determined by TGA (10 °C/min). ^b^ Determined by DSC (10 °C/min, 2nd heating scan). ^c^ Baseline shift assignable to glass transition was not observable.

A possible reason is the π-π stacking of the benzene rings in both of the components, by which the interaction between BZn and PS was strengthened in a similar manner with hybrid materials of PS and silica materials bearing aromatic rings [21,22]. Clear baseline shifts in DSC thermograms assignable to glass transition were observed only for BZn/PS. The glass transition temperatures (*T*_g_) of BZn_10_/PS_90_ and BZn_20_/PS_80_ were 86 and 82 °C, respectively. The single *T*_g_s correlated with the weight composition support that BZn and PS are miscible. Although baseline shift was not observed for TZn and the composite films, the transparency of the films implies that TZn is miscible with PMMA and PS. A plausible reason for the absence of the baseline shift is the decomposition took place before glass transition.

#### 2.3.2. Refractive Properties of Hybrid Polymers

**Figure 4:**
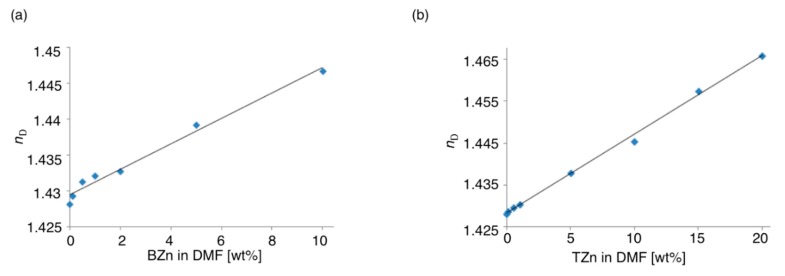
Concentration dependences of *n*_D_ of DMF solutions of (**a**) BZn; and (**b**) TZn.

We tried the measurement of the refractive indices of the films using an Abbe refractometer. However, the films were dissolved in contact liquids (bromonaphthalene and diiodomethane), and hence, we could not measure the refractive properties of the films. Accordingly, we estimated the refractive properties with the concentration dependence of the refractive indices (*n*_D_) of the zinc hybrid polymers in DMF. The *n*_D_ values of the DMF solutions of BZn and TZn increased linearly with the concentration (Figure 4). We estimated the *n*_D_ values of BZn and TZn as 1.60 and 1.61, respectively, via extrapolation of these linear relationships. The *n*_D_ value of the trithiol from TDT and EHA estimated in this method was 1.53, namely the hybridization with zinc increased the *n*_D_ value by 0.08. These *n*_D_ values are higher than those estimated for the zinc hybrid polymer (1.58) prepared from TDT and octadecylamine prepared via the previous method owing to the shortened alkyl chains [11]. The *n*_D_ values of these polymers are higher than typical commercial optical polymers like poly(ethylene terephthalate) (*n*_D_ = 1.57–1.58) and polycarbonate (*n*_D_ = 1.58–1.59), manifesting the effectiveness of this concept employing sulfur as the bridge between organic and inorganic components.

## 3. Experimental Section

### 3.1. Materials

Dehydrated THF (Kanto Chemical, Tokyo, Japan), DMF (Kanto Chemical), and DMSO (Kanto Chemical) were stored under a nitrogen atmosphere, and used as received. EHA (Tokyo Chemical Industry, Tokyo, Japan) was distilled under a nitrogen atmosphere. Zn(OAc)_2_ (Aldrich, St. Louise, MO, USA) was used as received. TDT was prepared via the reaction of tris-(2,3-epoxypropyl)isocyanurate, kindly supplied by Nissan Chemical Industries (Tokyo, Japan), and CS_2_ (Kanto Chemical) as reported [18]. BPA5DT was prepared via the reaction of bisphenol-A diglycidyl ether (Tokyo Chemical Industry) and CS_2_ as reported [16].

### 3.2. Measurements

^1^H-NMR spectra were measured on an ECX-400 instrument (JEOL, Tokyo, Japan) using tetramethylsilane as an internal standard (400 MHz). Fourier transform infrared spectra were measured on a FT-210 instrument (Horiba, Kyoto, Japan). Size exclusion chromatography measurements were performed on a HLC-8220 GPC (Tosoh, Tokyo, Japan) equipped with Tosoh TSK-gel superAW5000, superAW4000, and superAW3000 tandem columns using THF with a flow rate of 1.0 mL/min as an eluent at 40 °C. Quantitative elemental analysis was performed with a system consisting of a JEOL JSM6510A scanning electron microscope equipped with a JEOL JED2300 EDX spectrometer operated at an acceleration voltage of 20 kV. The samples were compressed as flat tablets, and the atom ratios were calculated as averages of data obtained from ten spots. Refractive indices (*n*_D_s) were measured with a DR-A1 digital Abbe refractometer (Atago, Tokyo, Japan).

### 3.3. One-Pot Synthesis of Organic-Sulfur-Zinc Hybrid via Sequential Ring-Opening Addition of EHA and TDT and Polycondensation of the in Situ Generated Thiol with Zn(OAc)_2_ (Typical Procedure)

TDT (105 mg, 199 µmol), EHA (77.3 mg, 599 µmol), and DMSO (5.0 mL) were added to a round-bottom flask, and the mixture was stirred at room temperature for 4 h under a nitrogen atmosphere. Then, a methanol solution (1.0 mL) of Zn(OAc)_2_ (56.4 mg, 307 µmol) was added to the mixture. The mixture was stirred at 30 °C for 24 h under a nitrogen atmosphere. The resulting solution was poured into an excess amount of methanol, and the precipitate was collected with filtration. The zinc hybrid polymer was obtained as white solid after washing with methanol and acetone followed by drying under reduced pressure (89.7 mg, 88.9 µmol, 45%). ^1^H-NMR (DMSO*-d*_6_/CF_3_CO_2_H = 5:1, δ in ppm): 0.6–0.9 (18H, C*H*_3_-), 0.9–1.3 and 1.3–1.8 (27H, CH_3_(C*H*_2_)_3_(CH_3_C*H*_2_)C*H*-), 2.5–3.8 (6H, -NC*H*_2_-), 3.0–4.3 (12H, -C*H*_2_CHC*H*_2_SZn), 5.2–6.0 (3H, -C*H*_2_CHC*H*_2_SZn), 8.8–9.3 (3H, -(C=S)N*H*CH_2_-).

### 3.4. One-Pot Synthesis of Organic-Sulfur-Zinc Hybrid via Sequential Ring-Opening Addition of EHA and BPADT and Polycondensation of the in Situ Generated Thiol with Zn(OAc)_2_ (Typical Procedure)

BPADT (97.1 mg, 197 µmol), EHA (55.2 mg, 427 µmol), and DMSO (5.0 mL) were added to a round-bottom flask, and the mixture was stirred at room temperature for 4 h under a nitrogen atmosphere. Then, a methanol solution (1.0 mL) of Zn(OAc)_2_ (37.1 mg, 202 µmol) was added to the mixture. The mixture was stirred at 30 °C for 24 h under a nitrogen atmosphere. The resulting solution was poured into an excess amount of methanol, and the precipitate was collected with filtration. The zinc hybrid polymer was obtained as white solid after washing with methanol and acetone followed by drying under reduced pressure (94.8 mg, 116 µmol, 59%). ^1^H-NMR (DMSO*-d*_6_/CF_3_CO_2_H = 5:1, δ in ppm): 0.6–0.9 (12H, C*H*_3_-), 0.9–1.8 (24H, CH_3_(C*H*_2_)_3_(CH_3_C*H*_2_)C*H*-, Ar-C-C*H*_3_), 2.6–3.1 (6H, -NC*H*_2_-), 3.1–4.4 (8H, -C*H*_2_CHC*H*_2_SZn), 5.4–5.8 (2H, -C*H*_2_CHC*H*_2_SZn), 6.5–6.9 and 6.9–7.2 (8H, -C_6_*H*_4_-), 7.9–8.3 (2H, -(C=S)N*H*CH_2_-).

### 3.5. Preparation of Composite Films (Typical Procedure)

PMMA and zinc hybrid polymer was added into a glass vial at the feed weight ratio of 10:1. Then, the mixture was dissolved in THF and the solution was poured into a Teflon mold. Films were obtained by gentle drying of THF at room temperature and drying under reduced pressure at 60 °C.

## 4. Conclusions

Soluble organic-sulfur-zinc hybrid polymers were prepared via one-pot and sequential reactions consisting of nucleophilic ring-opening addition of an amine to multi-functional cyclic dithiocarbonates and subsequent polycondensation of *in situ* generated thiols with Zn(OAc)_2_. The one-pot method overcame a problem that long octadecyl chains were necessary for solubility, and allowed the use of 2-ethylhexylamine instead. As a result of the shortened alkyl chains, the calculated *n*_D_ values of the hybrid polymers, 1.61 and 1.60, were higher than that of the previous product bearing octadecyl chains. The hybrid polymers may be cast as transparent films in the presence of PMMA or PS, and are potentially applicable as refractive ingredients. The results obtained in this work expanded the possibility of organic-sulfur-organic hybrid materials as optical materials.
